# Consequences of Different Mechanical Surface Preparation of Ni-Base Alloys during High Temperature Oxidation

**DOI:** 10.3390/ma13163529

**Published:** 2020-08-10

**Authors:** Wojciech J. Nowak, Krzysztof Siemek, Kamil Ochał, Barbara Kościelniak, Bartek Wierzba

**Affiliations:** 1 Department of Materials Science, Faculty of Mechanical Engineering and Aeronautics, Rzeszow University of Technology, Powstanców Warszawy 12, 35-959 Rzeszów, Poland; kochal@prz.edu.pl (K.O.); b.koscielnia@prz.edu.pl (B.K.); bwierzba@prz.edu.pl (B.W.); 2 Joint Institute for Nuclear Research, 141980 Dubna, Russia; siemek.krzysztof@gmail.com; 3 Institute of Nuclear Physics Polish Academy of Sciences, 31-342 Krakow, Poland

**Keywords:** Ni-base alloys, oxidation resistance, surface roughness, oxidation kinetics, oxide scale formation, residual stresses, defects

## Abstract

The influence of surface roughness on its high temperature oxidation for an Ni-base superalloy was studied using laser profilometry, atomic force microscopy, mass change measurements, glow-discharge optical emission spectrometry, scanning electron microscopy, X-ray diffraction, and positron annihilation methods. The isothermal and cyclic air oxidation tests were performed at 1000 °C and showed dependence of oxidation behavior on surface roughness. Smoother surfaces oxidation resulted in the formation of a multilayered oxide scale consisting of NiO, Cr_2_O_3_, and internally oxidized Al_2_O_3_ while a rougher surface formed protective Al_2_O_3_ scale. The factors responsible for different oxidation behaviors were determined as higher concentration of vacancies and increased residual stresses in the near-surface region of studied alloys.

## 1. Introduction

The materials used for the production of machinery elements working at high temperature, e.g., in gas turbines of jet engines or stationary gas turbines, have to fulfil a number of outstanding properties such as suitable ductility at low temperature, high creep strength, and high oxidation resistance over a wide range of operating temperatures, environments, and loading conditions. One type of material meeting such requirements is the Ni-base superalloys family. Using metallic alloys at high temperature results in their degradation due to high temperature corrosion, mainly by the reaction between the metal and oxygen [1]. Therefore, the high temperature oxidation resistance of used alloy is of major importance in terms of the component lifetime. In general, according to Giggins and Pettit [2], the alloys can be classified into three types, namely NiO, Cr_2_O_3_ (chromia), and Al_2_O_3_ (alumina) forming alloys. Considering the oxidation kinetics, the most protective oxide scale is an alumina scale [3]. Then, to elongate the real lifetime of gas turbine components, the highest focus is put to force the alloy to form protective alumina scale. One of the ways to promote alumina scale formation is an increase of Al-content in the alloy [4]. However, there is a limit of Al-content in Ni-base superalloys. Therefore, a further step to provide the corrosion resistance is the manufacturing of Al-rich coatings, such as e.g., an aluminide coating [5,6,7,8], or MCrAlY-type coatings [9,10,11,12]. However, very good effectiveness, in terms of increasing in oxidation resistance by applying protective coatings, demands the additional time and increases the total cost of the components. Therefore, an alternative and cheaper way for improving oxidation resistance of Ni-base superalloys is needed. Several attempts involving the role of surface roughness on high temperature oxidation behavior were taken by many different authors, i.e., for In-Ag alloy [13], Zircalloy-4 [14], pure Fe and Fe-based alloys [15,16,17,18], pure Cu and Cu-Ni alloys [19,20], Ni-Fe alloys [21], as well as for Ni-base alloys [22,23,24,25]. The findings showed contradictory effects of surface roughness on oxidation resistance depending on alloy chemistry, exposure temperature or type of the alloy. In few works, a mechanism responsible for the effect of surface roughness was proposed. Pei et al. [25] studied (Ni-base single crystal) DD6 superalloy with different surface roughness and found an increasing Al diffusion flux from the alloy to the surface for the roughest surface, resulting in the formation of a protective alumina scale. However, no exact factor responsible for higher Al-flux was given. Nowak et al. [23] manifested the hypothesis that rougher surface preparation method results in introduction of higher amount of point defects or their clusters into the near-surface region which act as easy diffusion paths for Al. However, no clear evidence for the introduction of such defects was shown. Moreover, in both mentioned works, the effect of surface roughness on oxidation kinetics was observed during short-term oxidation tests. The aim of the present work is for the first time to elucidate the factors responsible for the change in oxidation behavior of selected Ni-base superalloy, namely Mar M 247, and the consequences of such a change during long-term exposure. Investigations were performed by complex studies including laser profilometry, atomic force microscopy, mass change measurements, glow-discharge optical emission spectrometry, scanning electron microscopy, X-ray diffraction, and positron annihilation methods.

## 2. Materials and Methods

In the present work, two types of material, namely the commercially available Ni-base superalloy Mar M 247 and Ni-base model alloy with the nominal compositions, given in Table 1, were investigated.

From the block of cast Mar M 247, rectangular specimens with the dimension of 20 × 10 × 2 mm^3^ were machined. After cutting, all specimens were ground up to 500 grit using sandpaper. Such surface preparation was the starting point for all tested specimens. Then, one set of specimens was ground using 80 grit sandpaper and another set was polished up to 1 µm diamond paste. After mechanical surface preparation, all samples were ultrasonically cleaned in ethanol and dried with compressed air. Then, the analysis of surface roughness was performed using Sensofar S-Neox Noncontact 3D Optical Profiler (Sensofar, Barcelona, Spain). After roughness evaluation, samples were cleaned once again and subjected to three types of oxidation tests, namely an isothermal oxidation, a rapid cyclic oxidation, and a slow cyclic oxidation. The isothermal oxidation test was performed using XERION X-tube vertical furnace made by Xerion Advance Heating Ofentechchnik GmbH (Freiberg, Germany). The applied heating rate was 10 K/min and the flow of synthetic air was fixed as 2 L/min at 1000 °C up to 48 h. The rapid oxidation test was performed in furnace SCZ 120/150 made by Czylok (Jastrzębie Zdrój, Poland) at 1000 °C. In this furnace the samples were hanged on Khantal hooks in the movable sample holder. Each cycle consists of 2 h heating and 15 min cooling with pressurized air. For heating period, the sample holder moves immediately into the hot zone of the furnace after the hot zone of furnace reaches set temperature. The temperature in the furnace is controlled by two pyrometers and additionally by a thermocouple placed in the center of sample holder. This means that the samples are moved for heating period directly to desired temperature and kept for 2 h at 1000 °C. After 2 h heating, the sample holder automatically moves out of the furnace into the cold zone and the samples are immediately cooled using pressurized air. The slow cyclic oxidation test was performed in XERION X-tube horizontal furnace made by Xerion Advance Heating Ofentechchnik GmbH (Freiberg, Germany). In the latter case, each cycle was consisted of 2.5 h heating period and 0.5 h cooling with fan. In this type of test, samples placed in the sample holder were located in quartz tube with the dry air flow of 2 L/min. A furnace is movable, and the sample holder stays at the same position. During heating, the furnace moves to the position where the sample holder is in the hot zone of furnace, while for cooling period furnace moves into the cold zone and fan cools the quartz tube automatically for 0.5 h. Then, the temperature changes are not as drastic as in case of rapid oxidation test. During the oxidation test the mass changes were measured after each cycle during cooling period. After the oxidation test, the selected samples were subjected to depth profiling using glow discharge optical emission spectrometer (GD-OES) made by Horiba Jobin Yvon (Longjumeau, France). GD-OES depth profiles were quantified according to procedure described elsewhere [26,27,28]. After depth profiling, the metallographic cross-sections were prepared according to standard procedure. The cross-sections were observed with light optical microscope Nikon Epiphot 300 (Nikon, Tokyo, Japan) and analyzed by scanning electron microscope Hitashi S3400N (Hitahi, Tokyo, Japan).

For evaluation of the factors participating in altering of the oxidation behavior observed on Mar M 247, a high purity (99.99%) model alloy Ni-14Cr-4Al was investigated to limit the effect of complex chemical composition of the alloy. Firstly, from the rod with 10 mm diameter, the coupons with 2 mm height were cut and ground up to 500 grit using sandpaper. As previously, such surface preparation was the starting point. After grinding, the samples were ultrasonically cleaned with ethanol and subjected to relaxation heat-treatment (RHT) to eliminate the residual stresses in prepared samples. For the heat-treatment, the samples were placed in alumina crucibles in furnace Carbolite STF 16/450 (Carbolite, Hope Valley, England) and flushed with 6N purity argon for 2 h to evacuate the oxygen from the atmosphere. After flushing, the furnace was heated up to 550 °C. When the furnace reached the set temperature, the tube with samples was moved into the hot zone of the furnace and kept for 6 h. After 6 h, the furnace was shut down and the tube was taken out that the samples were in the cold zone of the furnace. Then, the samples were cooled down to room temperature, the argon flushing was stopped, and the samples were taken out. Such heat-treated samples were subjected to residual stresses measurement using X-ray diffraction performed on a PROTO iXRD COMBO produced by Proto Manufacturing Ltd. (Toronto, ON, Canada). The parameters necessary for residual stresses measurement (Young modulus, Poisson ratio etc.) were adapted from commercial alloy Rene 80 with the Cr and Al content (14 and 3 wt.% respectively) [29] similar to investigated Ni-14Cr-4Al. After residual stress measurement, the samples were subjected to five different surface preparation methods, namely using 80, 220, 500, and 1000 grit sandpapers and polishing using 1 µm diamond paste. Such prepared samples were subjected for surface roughness evaluation using Atomic Force Microscope (AFM) Naio AFM (Nanosurf, Lestal, Switzerland). The AFM investigation was carried out using static force mode under standard atmospheric conditions. The area with the dimensions 66 × 66 µm^2^ was scanned by 256 lines with scanning speed 1.2 s per line. The samples with these five differently prepared surfaces were subjected once again for residual stresses measurement to evaluate the effect of surface preparation method on stress situation in the near-surface region of the samples. The selected samples (polished and ground up to 80 grit sandpaper) were analyzed using positron annihilation methods: positron lifetime and Doppler broadening of annihilation line spectroscopy. The first method-positron lifetime measurements were done using equipment with timing resolution equaled 180 ps based on digital spectrometer APU-8702RU with BaF_2_ scintillators detectors (TechnoAP Co., Mawatari, Ibaraki, Japan). In this experiment, a positron ^22^Na source with activity 27 µCi enclosed into two 5-µm thick titanium foils was placed between two similarly prepared samples. Obtained spectra (contained 2 mln. counts) and was analyzed using LT 9.2 program [30]. During the deconvolution of the source contribution, background and finite time resolution were taken into account as adjustable parameters. The source contribution was equal 19% and consisted of two components, the first one 242 ps (with intensity 97%) and the second one 952 ps (3%).

Doppler broadening of annihilation spectroscopy measurements was performed using similar built ^22^Na source with activity 15 µCi. The HPGe detector ORTEC GEM25P4-70 (ORTEC, Oak Ridge, TN, USA) with an energy resolution (FWHM) of 1.20 keV at 511 keV was used to determine an annihilation line shape parameter S. It is defined as the number of counts below the central part of annihilation peak to the total number of counts in the range of this line, after background subtraction. The S parameter value is linked with the fraction of positron annihilation with low momentum electrons. Such electrons are mainly present in open-voids structural defects like for example vacancies and its clusters, so S parameter increases when the defects are present in sample. However, the dependency between its value and defects’ concentration is not linear and can also be sensitive to the type of defects. The positron implantation range emitted from 22 Na in material is limited, for pure iron is around 27 µm. To get information about changes of S parameter in the dependency on depth, the samples were successively chemically etched in aqua regia. In this way, a layer of a few micrometers was removed, and measurement was repeated. In other words, the depth profile was revealed. The etching does not introduce additional defects. The Positron Anihillation Spectrometry PAS measurements combined with etching experiments were chosen due to the fact that previously it gave interesting results in the field of defect studies for plastically deformed materials [31,32,33,34].

## 3. Results

The work consists of studies on two materials, namely, commercial Ni-base superalloy Mar M 247 and high purity model alloy. Investigation of commercial Ni-base superalloy was performed to illustrate the effect of surface preparation method on its oxidation behavior during high temperature exposure while high purity ternary Ni-Cr-Al model alloy was used to elucidate the factors responsible for different oxidation behaviors observed on commercial alloy.

### 3.1. Oxidation of Commercial Ni-Base Superalloy MAR 247

#### 3.1.1. Surface Roughness Description

**Figure 1:**
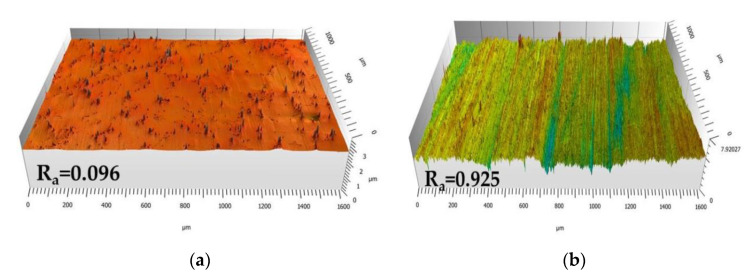
Three-dimensional reproduction of surface of Mar M 247 after: (**a**) Polishing up to 1 µm diamond paste; (**b**) Grinding using 80 grit sand paper performed using laser profilometer.

Figure 1 shows the three-dimensional reconstruction of the surface topography of Mar M 247 after polishing using 1 µm diamond paste and grinding using 80 grit sandpaper (Figure 1a,b respectively). The investigated areas of the surface were exactly the same (1600 × 1000 µm^2^) then one should notice that the values on *Y*-axis are different, i.e., on representation of ground surface value on *Y*-axis is bigger. This means that grinding process results in bigger surface roughness. The latter is confirmed by calculation of average roughness parameter R_a_ which for ground surface is one order of magnitude higher than for polished surface (0.925 and 0.096 µm respectively). Such observation is in a good agreement with findings published in previous work [18]. Regarding the nature of surface preparation, the scratches are observed on surface, which are additionally preferentially oriented. Then, to avoid the influence of anisotropy on surface roughness parameters, R_a_ values were calculated using the profile taken in a direction perpendicular to the grinding direction.

#### 3.1.2. Short-Term Isothermal Air Oxidation

**Figure 2:**
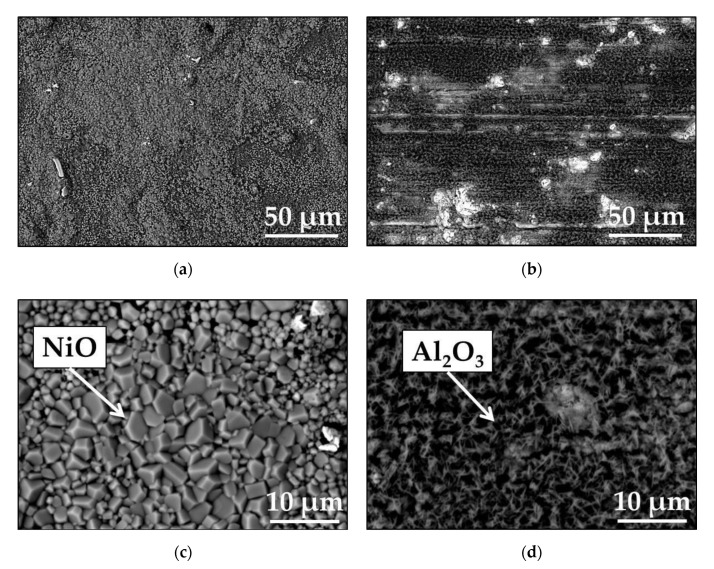
Scanning electron microscopy backscattered electron SEM/BSE images performed on surface of Mar M 247 after isothermal air oxidation test at 1000 °C up to 48 h showing oxide scale formed on: (**a**) polished up to 1 µm diamond paste taken at low magnification (**a**) and high magnification (**c**); (**b**) ground using 80 grit sand paper taken at low (**b**) and high magnification (**d**).

The scanning electron microscopy backscattered electron SEM/BSE images of the surfaces of polished and ground Mar M 247 after isothermal air oxidation tests are shown in Figure 2. As visible, (Figure 2a,b) in case of polished surface, formed oxide scale is rather uniform and consists of regular grains, while for ground surface, a heterogeneous oxide scale can be observed. Moreover, an indication of grinding direction can be seen in Figure 2b in form of scratches. The images taken at higher magnification in combination with electron diffraction spectrometry SEM/EDS analysis revealed that most surface of polished sample is covered with NiO, while on ground surface mostly Al_2_O_3_ oxide scale is developed. The spiky shape of Al_2_O_3_ suggests that the Al_2_O_3_ formed as θ-Al_2_O_3_ (see Figure 2d).

**Figure 3:**
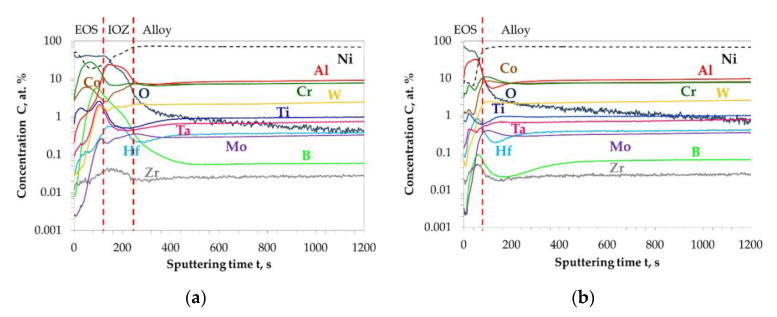
GD-OES depth profiles obtained on Mar M 247 after isothermal air oxidation at 1000 °C up to 48 h with: (**a**) Polished surface; (**b**) Ground surface. The red dashed lines virtually indicate the regions of external oxide scale (EOS), internal oxidation zone (IOZ) and metallic alloy.

GD-OES depth profiles showing the elements concentration distribution as function of sputtering time obtained on polished and ground Mar M 247 after isothermal air oxidation at 1000 °C for 48 h are shown in Figure 3a,b, respectively. In GD-OES depth profile obtained on polished alloy (Figure 3a) three zones can be distinguished, namely: an external oxide scale (EOS), an internal oxidation zone (IOZ) and a bulk alloy zone. One can see that during the very first seconds (0 to 30 s) of sputtering, a co-enrichment of Ni and O is found. At later stage of sputtering (30 to 120 s), an increase in Cr and Co content is observed. After Co/Cr co-enrichment, a drop in O content and a clear increase in Al content is found for sputtering time between 120 and 210 s. Between the Co/Cr co-enrichment and Al enrichment, the increase in content of the elements like B, Ti, Ta is observed. One should notice that the increase in B concentration is from 0.05 at.% (in the alloy region) to 5 at.% (for around 100 s of sputtering in Figure 3a). In contrast, for the GD-OES depth profile obtained on ground Mar M 247, after isothermal air oxidation, the absence of IOZ is noted. As visible in Figure 3b, a strong enrichment in Al and O is present for sputtering time from 0 to 60 s in the EOS. Around 60 s of sputtering, a co-enrichment of Hf and B is found, however the extent of enrichment of B is far less as compared to polished specimen. For both investigated surfaces, a depletion zone is observed in the alloy for the elements enriched in the oxide scale. A depletion zone can be identified by the lower element concentration as compared to the content in the alloy at later stages of sputtering, e.g., for a sputtering time longer than 1000 s.

**Figure 4:**
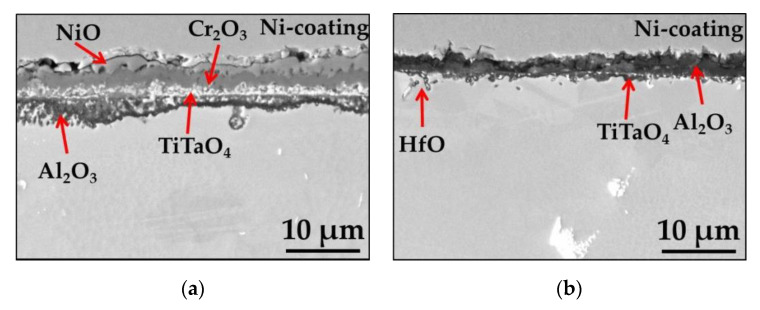
SEM/BSE images performed on cross-sections of Mar M 247 after isothermal air oxidation test at 1000 °C up to 48 h showing oxide scale formed on: (**a**) Polished surface; (**b**) Ground surface.

The SEM/BSE images of the cross sections of polished and ground Mar M 247 after isothermal air oxidation at 1000 °C for 48 h are shown in Figure 4a,b, respectively. The combination of SEM/EDS point analysis, GD-OES depth profiling, X-ray diffraction XRD results, and literature data allow for the identification of formed oxide scales. As shown in Figure 4, formation of NiO in the very outer part of the oxide scale is found on polished sample. Beneath NiO layer, Cr_2_O_3_ oxide containing minor amount of Co is formed. At the bottom of Cr_2_O_3_ layer, oxides containing Ti and Ta are found. Based on literature data, these oxides were identified as TiTaO_4_ [35]. Below the external oxide scale, a zone of internal oxidation of Al is also found, in which internally oxidized Al_2_O_3_ pegs are found together with remaining alloy between them (Figure 3a). On the contrary, a thin Al-rich oxide scale developed as external oxide scale on sample with ground surface (Figure 3b). Beneath EOS, the formation of internally oxidized pegs of HfO is observed as well as fine precipitates of Ti/Ta-rich oxides identified as TiTaO_4_. However, it should be mentioned that Ti/Ta-rich oxide precipitation is more prone to form as compared with ground one (compare thickness of bright precipitates on the cross-sections in Figure 3a,b) on polished alloy.

#### 3.1.3. Rapid Cyclic Oxidation Test

**Figure 5:**
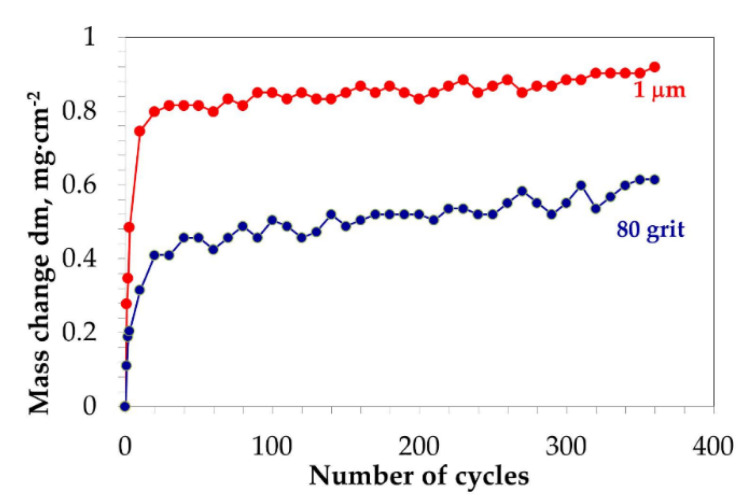
The mass change per unit area plots measured for polished and ground samples of Mar M 247 during rapid cyclic oxidation test. The cycles consisted of 2 h heating at 1000 °C and 15 min cooling with pressurized air.

**Figure 6:**
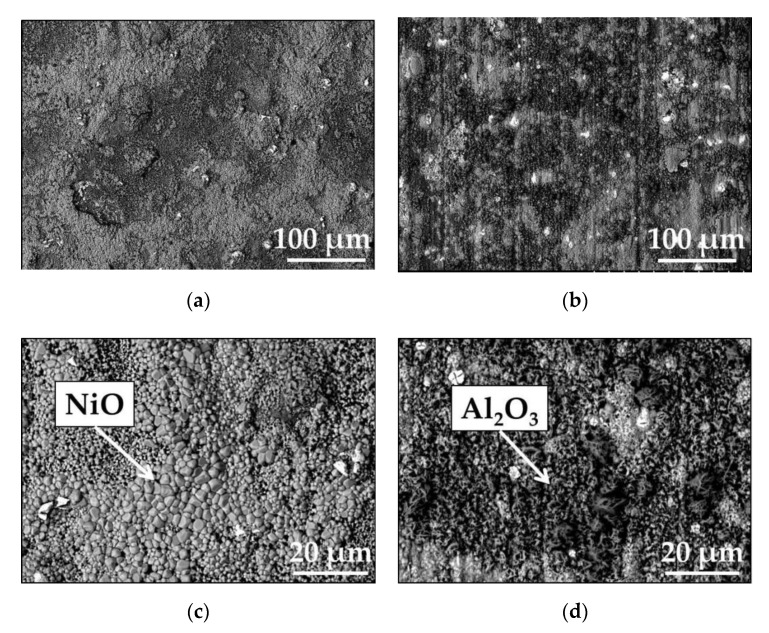
SEM/BSE images performed on surface of Mar M 247 after rapid cyclic oxidation test at 1000 °C after 360 cycles with: (**a**) polished up to 1 µm diamond paste taken at low magnification (**a**) and high magnification (**c**); (**b**) Ground using 80 grit sand paper taken at low (**b**) and high magnification (**d**).

**Figure 7:**
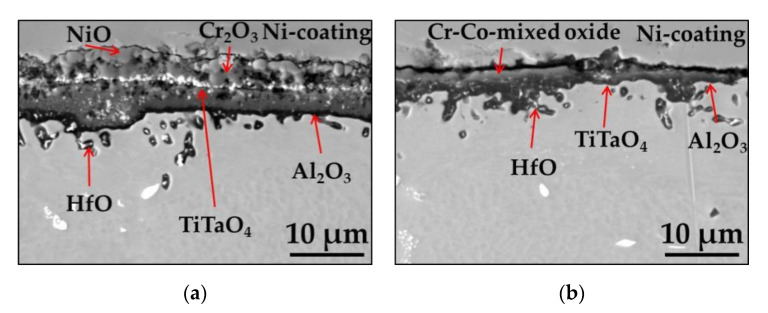
SEM/BSE images performed on cross-sections of Mar M 247 after rapid cyclic oxidation test at 1000 °C after 360 cycles with: (**a**) Polished surface; (**b**) Ground surface.

To elucidate the impact of different surface preparation method on alloy performance at high temperature, the cyclic oxidation tests were performed. The mass change measured during rapid cyclic oxidation tests showed that the alloy with polished surface revealed almost two times higher mass change than with ground surface (Figure 5). The significant difference in mass change occurred after 10 cycles (20 hot hours) of exposure and this trend is visible at the end of rapid cyclic oxidation test. The mass change per unit area of the polished sample at the end of the test was around 0.9 mg·cm^−2^, while for ground one about 0.5 mg·cm^−2^ (see Figure 5). The SEM/BSE images of the surface of oxidized alloys revealed similar phenomenon as observed after isothermal oxidation, namely majority of polished surface was covered by NiO, while on the ground surface, mainly Al_2_O_3_ was formed (Figure 6a–d). The SEM/BSE images of the cross-section of the alloys after rapid cyclic oxidation tests for 360 cycles (720 hot hours) (Figure 7a,b) revealed similar microstructure as observed after isothermal oxidation test, however, in case of alloy with polished surface after rapid cyclic oxidation test, a virtually continuous Al_2_O_3_ sub-scale can be observed together with formation of HfO pegs below mentioned continuous sub-layer (Figure 7a). Moreover, on the cross-section of ground alloy, a presence of Cr-Co mixed oxide is found. The arrows show the location of prescribed oxides.

#### 3.1.4. Slow Cyclic Oxidation Test

**Figure 8:**
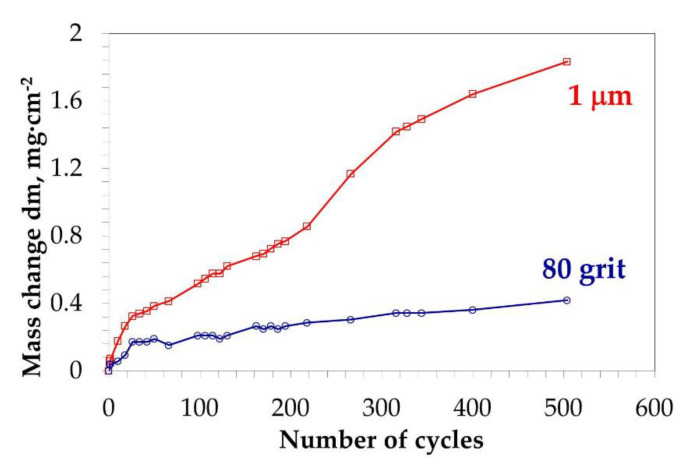
Normalized mass change plots measured for polished and ground samples of Mar M 247 during slow cyclic oxidation test. The cycles consisted of 2.5 h heating at 1000 °C and 0.5 h cooling.

**Figure 9:**
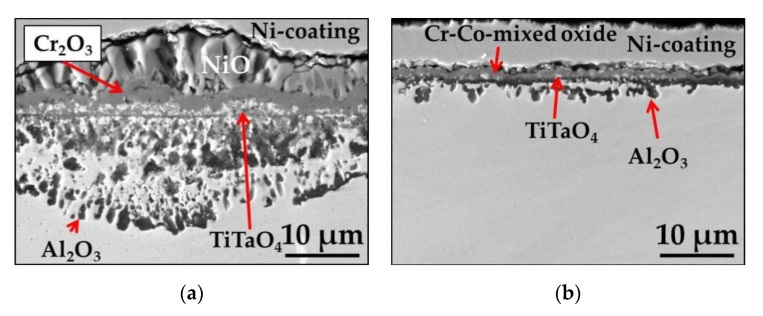
SEM/BSE images performed on cross-sections of Mar M 247 after slow cyclic oxidation test at 1000 °C after 500 cycles with: (**a**) Polished surface; (**b**) Ground surface.

To investigate the effect of cycles conditions on observed effect, a slow cyclic oxidation test was performed on polished and ground Mar M 247 for 500 cycles (1250 hot hours). As visible in plot depicted in Figure 8, the measured mass changes up to 200 cycles are similar to observed during rapid oxidation test, namely, for polished alloy higher mass change is observed than for ground one. After 200 cycles (500 hot hours) the measured mass change for polished alloy was about 0.8 mg cm^−2^ while for ground one around 0.3 mg cm^−2^. Only a slight increase in mass change for ground alloy was observed and at the end of test-the measured mass change was about 0.4 mg cm^−2^. Interestingly, shortly after 220 cycles (550 hot hours) sharp increase in mass change is observed for polished alloy. A total mass change at the end of the test obtained for polished specimen was about 1.8 mg cm^−2^. SEM/BSE images of the cross sections of polished and ground alloy after slow oxidation test (Figure 9a,b) revealed similar oxide scale microstructure as observed after isothermal and rapid oxidation tests. It should be noticed that differently for rapid cyclic oxidation, no continuous Al_2_O_3_ sub-scale is observed for polished sample but zone of internal oxidation of Al (see Figure 9a). Moreover, a thicker NiO layer on the top surface is found for the polished sample after slow cycling than the rapid one. Similar to the observation during the rapid oxidation test, the formation of Cr-Co mixed oxide on top of the ground surface is also found in the present case.

### 3.2. Oxidation of Model Alloy Ni-14Cr-4Al

**Figure 10:**
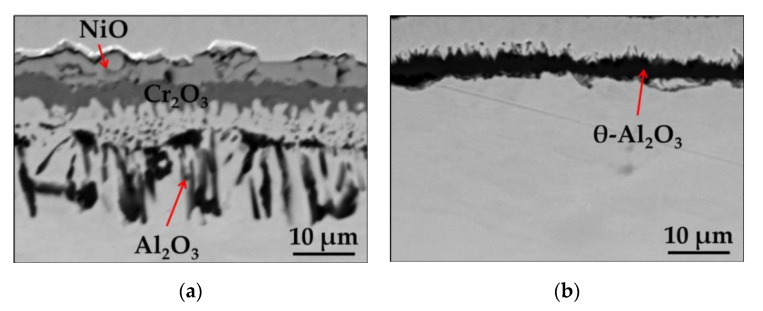
SEM/BSE images performed on cross-sections of Ni-14Cr-4Al model alloy after isothermal air oxidation test at 1000 °C for 24 h with: (**a**) Polished surface; (**b**) Ground surface.

To simplify the situation and reduce the parameters potentially influencing the oxidation behavior of Ni-base alloy, like e.g., complex alloy chemical composition, a ternary model alloy was investigated to elucidate the major parameters responsible for observed changes in oxidation behavior of Ni-base alloys. To check the reproducibility of observed effect of surface preparation method on formed oxide scale morphologies, an isothermal air oxidation test at 1000 °C for 24 h was performed. The SEM/BSE images of the cross-sections of polished and ground Ni-14Cr-4Al model alloy after isothermal air oxidation (Figure 10a,b) revealed a similar effect as observed for commercial alloy. Namely, there is an oxide scale consisting of NiO in the very outer part of the oxide scale, below which Cr_2_O_3_ layer and zone of internally oxidized Al_2_O_3_ was found on polished alloy (Figure 10a), while a relatively thin Al_2_O_3_ oxide scale formation is visible on ground alloy (Figure 10b). The spiky shaped morphology of formed Al_2_O_3_ suggests a formation of metastable θ-Al_2_O_3_. Then, the similar effect of surface preparation method on oxide scale morphology is observed for the commercial as well as model alloy.

### 3.3. Consideration about Factors Altering Oxidation Behaviour of Ni-Base Alloys

To find out the factors responsible for such differences in oxidation behavior of Ni-base alloys, a series of different surface preparation methods were applied for Ni-14Cr-4Al model alloy after relaxation heat treatment. Namely, five different surface preparation methods were done: polishing up to 1 µm diamond paste, and grinding using 1200, 500, 220, and 80 grit sandpapers. The samples prior and after surface preparation were subjected for surface roughness evaluation, residual stress measurement, and selected samples were investigated by positron annihilation.

#### 3.3.1. Surface Roughness

**Figure 11:**
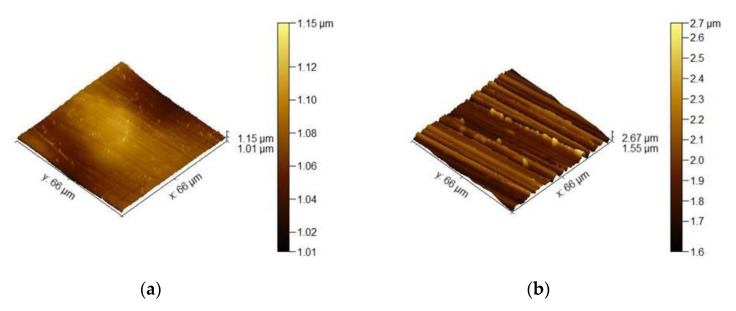

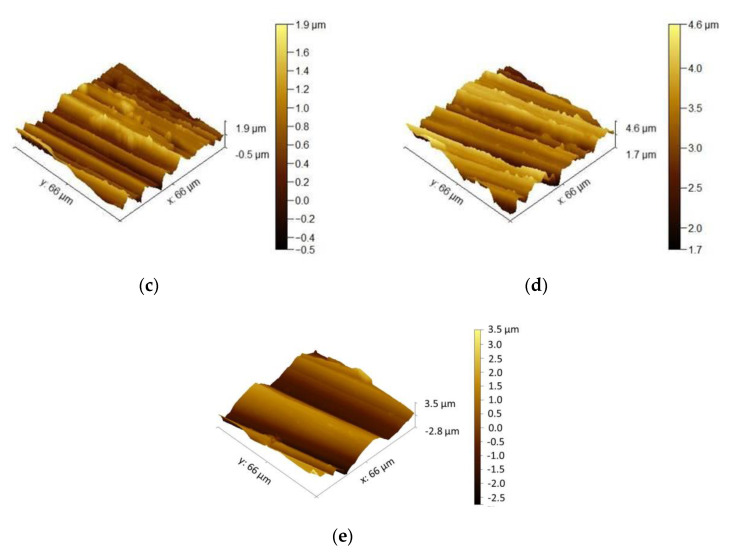
Images showing surface topography of Ni-14Cr-4Al model alloy with: (**a**) polished up to 1 µm diamond paste; (**b**) Ground using 1200 grit sand paper; (**c**) Ground using 500 grit sand paper; (**d**) Ground using 220 grit sand paper; (**e**) Ground using 80 grit sand paper.

**Figure 12:**
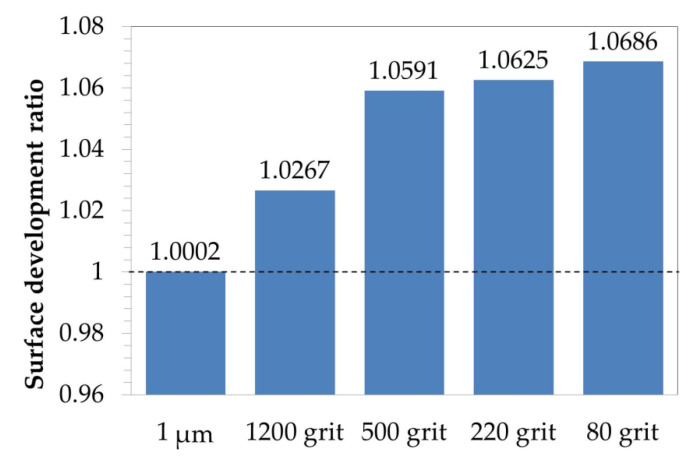
Plot showing surface development ratio referred to an ideally flat surface calculated based on measurement by AFM from Figure 11 for Ni-14Cr-4Al model alloy with different surface preparation method.

Figure 11 shows the reproduction of surface topography of differently prepared Ni-14Cr-4Al alloy. As shown, the decreasing of sandpaper gradation obviously results in rougher surface. The surface topography reproduction was made using atomic force microscopy on region with dimensions 66 × 66 µm^2^. One should notice that despite increasing waviness of surface (deeper scratches) also value on *Y*-axis is increasing. Using 3D data of surface topography reproduction, a surface development ratio was calculated and shown in Figure 12. Surface topography ratio was calculated by dividing the actual, measured surface area by projected, ideally flat surface (66 × 66 µm^2^). The dashed line shown in Figure 12 illustrates ideally flat surface. The calculated results show that a polished surface is almost ideally flat, while the decreasing of sand paper gradation increases the surface development ratio.

#### 3.3.2. Residual Stresses

**Figure 13:**
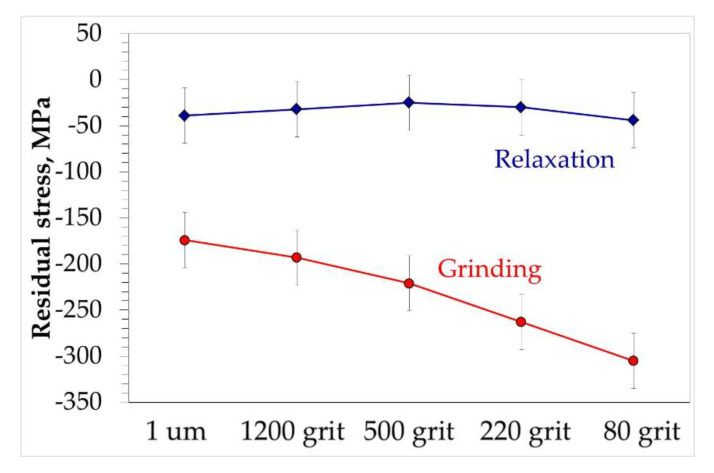
Plot showing measured residual stresses on Ni-14Cr-4Al samples at stage after relaxation heat-treatment and after different mechanical surface preparation method.

To evaluate the change in residual stresses in the material caused only by different mechanical surface preparation method, the samples prepared of model alloy were subjected to relaxation heat treatment at 550 °C for 6 h to eliminate the stresses caused by casting of rods. To ensure that the measurement of residual stresses after RHT and after surface preparation was performed exactly on the same sample each sample was marked and the residual stress measurement has been performed on each of unequivocally marked samples. The obtained results are shown in Figure 13 as blue line called “relaxation”. Despite the fact that the measured residual stresses after RHT are shown as a function of surface preparation, it should be clearly highlighted that no mechanical surface preparation was applied. Points shown in blue “Relaxation” curve represent the residual stress values obtained after relaxation heat treatment stage. Showing this curve as function of surface treatment was meant to show the procedure of residual stress measurement. Namely, each sample after RHT was marked and designated to specific mechanical surface preparation. Then, it was ensured that the residual stress measured after RHT and after surface preparation was performed exactly on the same specimen. It is visible that the stress level is approximately uniform, and it is at level of −50 MPa. After residual stresses measurement in RHT stage the samples were subjected for prescribed surface preparation and the residual stresses were measured once again in the as-prepared stage. The results obtained after surface preparation are depicted in Figure 13 by a red line described as “grinding”. The results shown in Figure 13 reveal that each surface preparation method results in increased compressive residual stress which rises with sandpaper gradation.

#### 3.3.3. Defects in the Near-Surface Region

To evaluate the theory proposed in the previous works [18,19,20,21] that the different mechanical surface preparation method introduces a different number of defects in the near surface region of the samples, selected samples were subjected to positron annihilation analysis. Two positron techniques were used. The first one is positron lifetime (LT) measurements. This analysis allows to get information about the kind of defects. In reference state only one positron lifetime component was obtained equal 122 ps. Its value is a little higher than value for non-defected pure nickel 109 ps and chromium 120 ps [36], which indicates on presence of jogs on dislocations edges for which positron lifetime is higher, i.e., for Ni is equal 119 ps [37]. After polishing with 1 µm diamond paste, no additional defects were created, and positron lifetime remains the same. The increment of positron mean lifetime is observed on samples after grinding with 80 grit sandpaper, see Table 2. For these samples two component analyses were performed. The shorter lifetime τ_1_ stands for dislocations and longer one τ_2_ = 230 ps indicates the presence of small vacancy clusters. The most similar lifetime value to measured one τ_2_ was calculated for pure Ni for four vacancies agglomeration and is equal 257 ps [37]. The performed studies show that different types of defect are produced during different surface treatments in NiCrAl alloy.

**Figure 14:**
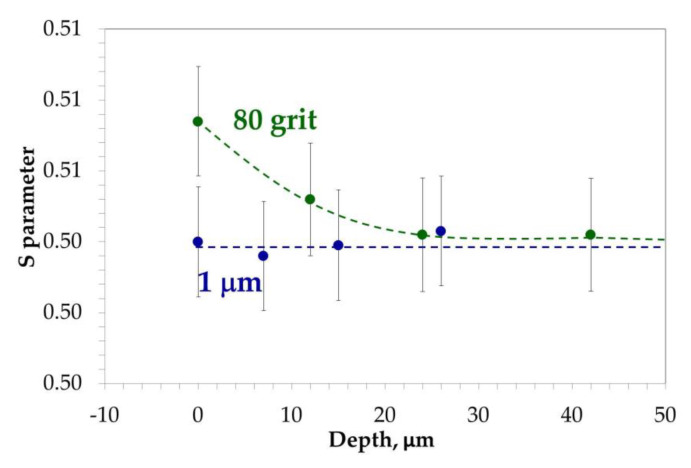
Plot showing measured S parameter as function of depth by positron annihilation method.

Positron Doppler spectroscopy experiment was performed sequentially with etching to study depth distributions of defects after different surface treatment, see Figure 14. According to assumptions, the sample polished with 1 µm particles gives a constant value of S parameter and does not reveal presence of any additional defects similar to the positron lifetimes measurements. For ground samples measured S parameter change was observed. Higher value of S parameter near surface was noted for ground samples and it decreases up to 30 µm to the level of value of reference sample. This means that defect concentration decreases with each etching step, and finally, over 30 µm, all defects produced during grinding are removed.

## 4. Discussion

The results obtained in the present work showed that surface preparation method resulting in different surface roughness, significantly affects the oxidation behavior of studied alloys. Moreover, the obtained results showed that the observed effect is present not only in the case of short-term isothermal air oxidation, but also during long term cyclic oxidation of alloys with commercial use. The analyses performed on high purity model alloys with simpler chemical composition than commercial alloy Mar M 247 showed that the effect of surface preparation on oxidation behavior is observed also for model alloys. An investigation of model alloys in the as prepared stage allowed the identification of three factors potentially responsible for observed phenomenon, namely surface development ratio, residual stresses, and introduced defects.

The results of surface development ratio showed that increase of surface roughness obviously results in increase of surface development ratio. The results showed that change from polishing using 1 µm diamond paste to grinding using 80 grit sandpaper results in increase of surface development ratio of about 7%. This means that the oxygen from the atmosphere has 7% higher area to adsorb and reacts at the very beginning of the oxidation process. The oxidation reaction can be described by the following Equation:

(1) $$\frac{1}{2}O_{2}+M=MO$$

**Figure 15:**
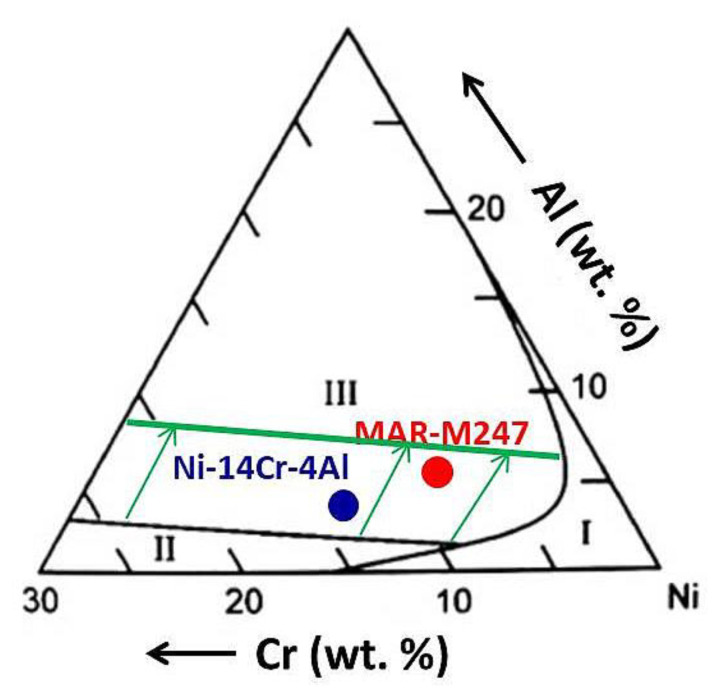
Oxidation map developed by Giggins and Pettit [2] with interpolated Cr and Al content for MAR 247 and Ni-14Cr-4Al alloys showing the effect of surface smoothening by polishing.

According to the Le Chatelier–Braun principle, the increase of the number of moles of oxygen available for the reaction will result in increase of moles of reaction products, i.e., MO [38]. This would result in increase of oxidation kinetics as increase of mass change curve. In the present study, the results showed the opposite effect, i.e., sample with smaller surface development ratio revealed higher oxidation kinetics. Therefore, the factor which overcompensates the higher surface development ratio for ground surface than polished should exist. One such factor can be the residual stress level. Surface preparation method resulting in higher surface roughness, causes higher compressive residual stresses in the near surface region. In the literature, a positive effect of compressive residual stresses on fatigue was previously described [39]. Apparently, as shown in the present work, the compressive residual stresses besides increasing of oxide scale adherence also affect the oxidation kinetics. Finally, the positron annihilation analysis confirmed proposed hypothesis about introduction of higher number of defects in the near surface region by rougher surface preparation method [23]. Apparently, the presence of defects also affects the residual stress level, i.e., for a sample with a higher number of defects, more compressive stress was also measured. It was claimed that present defects in the near surface region can act as an easy diffusion path for elements forming protective oxide scale at very early stages of oxidation. This can be done either by the diffusion of the aluminum via defects themselves or after recrystallization process, via grain boundaries of recrystallized grains [18]. According to Wagner’s theory, there are two criteria to form and maintain a continuous oxide layer [40]. First criterion is about transformation from internal to external oxidation, which for the case of Ni-Al alloys is:

(2) $$N_{Al}^{\left(1\right)}>{\left[\frac{\pi g^{*}}{3}N_{0}^{\left(S\right)}\frac{D_{0}V_{m}}{{\tilde{D}}_{Ni-Al}V_{AlO_{1.5}}}\right]}^{1/2}$$

where $N_{0}^{\left(S\right)}$ is the oxygen solubility in the alloy, $D_{0}$ is the diffusivity of oxygen in the alloy, ${\tilde{D}}_{Ni-Al}$ is the alloy interdiffusion coefficient, and $V_{m}$ and $V_{AlO_{1.5}}$ are the molar volumes of the alloy and the aluminium oxide, respectively. The factor $g^{*}$ is generally approximated as about 0.3 [13]. The second criterion concerns the situation, when continuous external scale is formed. In this case, diffusion in the alloy must be rapid enough to supply the solute at least at the rate at which is being consumed by scale growth [41]. In this case, the second criterion is:

(3) $$N_{Al}^{\left(2\right)}=\frac{V_{m}}{32_{\nu }}{\left(\frac{\pi k_{p}}{{\tilde{D}}_{Ni-Al}}\right)}^{1/2}$$

where $k_{p}$ is the parabolic rate constant for external alumina scale. The results obtained in the present work showed that the type of the oxide scale formed during the transient oxidation stage is detrimental for oxidation kinetics. It was shown that if at the beginning a multilayered oxide scale consisting of NiO, Cr_2_O_3_ and internally oxidized Al_2_O_3_ will be formed, then during long term cyclic oxidation a higher mass change will be observed than in case of the sample on which protective Al_2_O_3_ will be formed since the beginning. It was stated that rougher surface increases Al-flux [25], then the factor ${\tilde{D}}_{Ni-Al}$ in Equation (2) increases. Based on the results obtained within the present work, it is postulated that increase of ${\tilde{D}}_{Ni-Al}$ is caused by introduction of defects in the near-surface region. Long-term exposure revealed that formation of protective alumina scale at early stage of oxidation lowers the inward flux of oxygen $D_{0}$ in such extent, that no breakaway of alumina scale is observed (see e.g., Figure 8) and the alloys form protective alumina scale due to fulfilling second Wagners’ criterion (Equation (3)). Moreover, the results obtained during long term slow cyclic oxidation showed that formation of virtually continuous Al_2_O_3_ sub-layer does not effectively protect the studied alloy. As observed on mass change plot for polished sample (Figure 8) after roughly about 250 cycles (625 hot hours), an increase in mass change is observed. This observation in combination with finding during rapid cyclic oxidation (formation of virtually continuous Al_2_O_3_ sub-scale) suggest that after longer exposure time, a breakaway of the alumina sub-scale occurred. Then, in the case of a polished surface, both Wagner’s criteria are not fulfilled. Higher mass change gives an information about thickening of the oxidation products. Then, in the studied case, the critical oxide scale thickness [42] leading to oxide spallation will be reached faster in the case of samples with polished surfaces than with ground ones. Oxide scale spallation results in direct contact of the depleted alloy surface with oxygen, which will result in even higher oxidation rate. Finally, repeated spallation and re-growth of the oxide scale [43] would result in thinning of components walls, therefore decreasing of mechanical properties of the whole component. According to the ternary oxidation map developed by Giggins and Pettit [2], both investigated alloys, namely commercial Mar M 247 as well as model alloy Ni-14Cr-4Al can be classified as alumina forming alloys (Figure 15) if only Ni, Cr, and Al are considered. The field (marked as I) represents the alloys forming external oxide scale consisting of external NiO oxide scale with precipitates of internally oxidized Cr_2_O_3_ and/or Al_2_O_3_ precipitates, field II shows the alloys forming external Cr_2_O_3_ oxide scale with precipitates of internally oxidized Al_2_O_3_, while field III represents the alloys forming external Al_2_O_3_ oxide scale [2]. Since both studied alloys are classified as “alumina” forming alloys (region III, Figure 15). Since for studied temperature (1000 °C) oxidation constant rate (K_p_) obtained for Cr_2_O_3_ formation is equal to 2.8 × 10^−6^ mg^2^·cm^−4^·s^−1^ [44] while for Al_2_O_3_ is 3.8 × 10^−8^ mg^2^·cm^−4^·s^−1^ [45] then it is obvious that Al_2_O_3_ is more protective oxide scale comparing to Cr_2_O_3_. Then, the formation of Al_2_O_3_ during exposure at high temperature is beneficial and desired. As shown by the obtained results, the different surface preparation method alters the alloy to be an “alumina” (region III) or “chromia” forming alloy. Then, the transition from region “III” to region “II” is observed. Therefore, only transition from internal to external oxidation of Al was observed. The latter is the main reason why only Al critical content was discussed while critical Cr-content was neglected. As shown in Table 1, the model alloy possesses higher amount of Cr, namely 14 wt.% as compared to Cr content in commercial alloy Mar M 247 (8.5 wt.%). This could potentially affect the obtained results. However, both alloys are classified to be marginal alumina forming alloys (Figure 15) and on both alloys, a similar effect of surface preparation method was found. Therefore, a possible effect different Cr content in both alloys was neglected. The results obtained in the present work showed that by the smoothening of investigated alloy surface roughness, the border between the alumina and chromia forming alloys is shifted in the region of higher Al-content (as shown in Figure 15). It was found that sample with rougher surface contains defects in the near-surface region. The presence of defects is claimed as factor influencing the oxidation behavior of studied alloys. Namely, higher defects concentration in the near-surface region of material resulted in formation of protective oxide scale. It was claimed that the diffusion coefficient for Al in Ni is increased by the presence of the defects. However, it is not clear whether the defect serves as an easy diffusion path itself or lowers also activation energy needed to recrystallization, which in turn results in higher grain boundaries concentration. Independently of the exact mechanism, the presence of defects causes the formation of more protective oxide scale.

## 5. Conclusions

The results obtained within the present work allowed to formulate the following conclusions:

1. Surface preparation method resulting in different surface roughness significantly influences the oxidation behavior of studied alloys. Namely, on a polished surface, a multilayered oxide scale was found consisting of NiO, Cr_2_O_3_, and internal zone of Al_2_O_3_, while on the ground surface, mainly protective Al_2_O_3_ formation was observed.
2. The observed effect was found not only during short-term exposure, but also during long-term cyclic exposure at rapid and slow cyclic conditions.
3. Surface roughness evaluation revealed an increase in surface development ratio with the rougher surface preparation method. However, this factor was claimed not to be responsible for the change in oxidation behavior of the studied alloys.
4. It was found that the surface preparation method resulting in higher surface roughness also introduces higher compressive stresses into the near-surface region of the studied material. The material with higher compressive stress formed more protective oxide scale. Then, a higher compressive stress value was claimed to be one of the factors responsible for a change in the oxidation behavior of studied Ni-base alloys.
5. It was confirmed that the sample with a rougher surface contains defects in the near-surface region. The presence of defects was claimed as a second factor influencing the oxidation behavior of the studied alloys, causing the formation of protective oxide scale.
6. It was shown that, by the simple surface mechanical preparation method, the alloy can be moved from the region of alumina formation into chromia forming alloys. Then, in the case of studied alloys, polishing showed a negative effect in terms of high temperature oxidation behavior.

## Figures and Tables

**Table 1:** Nominal chemical composition of investigated alloys in the as-cast condition (given in wt.%).

Elements (wt.%)	Ni	Cr	Ta	Co	Mo	W	Al	Ti	Zr	B	Fe	Hf	C
Mar M 247	Base	8.5	3	20	0.7	10	5.5	1	0.05	0.02	0.16	1.5	0.14
Model alloy	Base	14	-	-	-	-	4	-	-	-	-	-	-

**Table 2:** Positron lifetime measured in 1 µm polished and 80 grit ground Ni14Cr4Al samples.

Sample Preparation	τ_1_ (ps)	I_1_ (%)	τ_2_ (ps)	I_2_ (%)	τ_mean_ (ps)
1 µm	122	100	-	-	122
80 grit	127	78.6	230	21.4	149
